# Integrative multi-omics analysis reveals inflammation-related molecular networks in acute mountain sickness

**DOI:** 10.3389/fimmu.2026.1745433

**Published:** 2026-05-22

**Authors:** Zhicheng Xiang, Haifeng Ma, Dahai Cao, Fei Zhang, Chunqing Yang, Taijian Cao, Qiang Zhang

**Affiliations:** 1 College of Clinical Medicine Qinghai University, Xining, China; 2 Department of Neurosurgery, Qinghai Provincial People’s Hospital, Xining, China; 3 Qinghai University Affiliated Hospital, School of Clinical Medicine, Qinghai University, Xining, China

**Keywords:** high-altitude cerebral edema, inflammation-related genes, monocyte-mediated inflammation, multi-omics integration, single-cell RNA sequencing

## Abstract

**Background:**

High-altitude cerebral edema (HACE) is a life-threatening condition following rapid ascent to high altitude, with acute mountain sickness (AMS) as a key precursor. Increasing evidence implicates inflammation in its pathogenesis, yet the molecular regulatory networks remain unclear.

**Methods:**

We integrated bulk RNA-seq (GSE75665), non-coding RNA-seq (GSE90500), and single-cell RNA-seq datasets to explore inflammation-related mechanisms in AMS. Differentially expressed genes (DEGs) were identified and analyzed using Gene Ontology (GO), Kyoto Encyclopedia of Genes and Genomes (KEGG), and Gene Set Enrichment Analysis (GSEA). Protein-protein interaction, miRNA-mRNA, transcription factor, and drug-gene networks were constructed. Single-cell and deconvolution analyses determined cell-type-specific expression and immune composition. A hypobaric hypoxia-induced HACE mouse model was established for experimental validation to assess heparin-binding EGF-like growth factor (HBEGF) expression and associated neuroinflammatory and pathological changes.

**Results:**

We identified 323 DEGs, including five 5 inflammation-related genes, all downregulated in AMS and enriched in epithelial cell growth, migration, and the ErbB signaling pathway. hsa-miR-375 was predicted to regulate multiple key genes, and drug gene analysis highlighted HBEGF as a potential therapeutic target. Single-cell data revealed monocytes as the major source of key gene expression and increased CD4^+^ monocytes in AMS. *In vivo*, HBEGF expression was significantly reduced in HACE mice, while Adeno-associated virus (AAV)-mediated HBEGF overexpression mitigated cerebral edema, blood-brain barrier disruption, neuronal injury, and proinflammatory cytokine release.

**Conclusions:**

This integrative multi-omics study identifies monocyte-mediated inflammation and the ErbB pathway as critical mechanisms in HACE. HBEGF emerges as a promising therapeutic target for preventing neuroinflammation and cerebral injury in high-altitude conditions.

## Graphical Abstract

**Figure:**
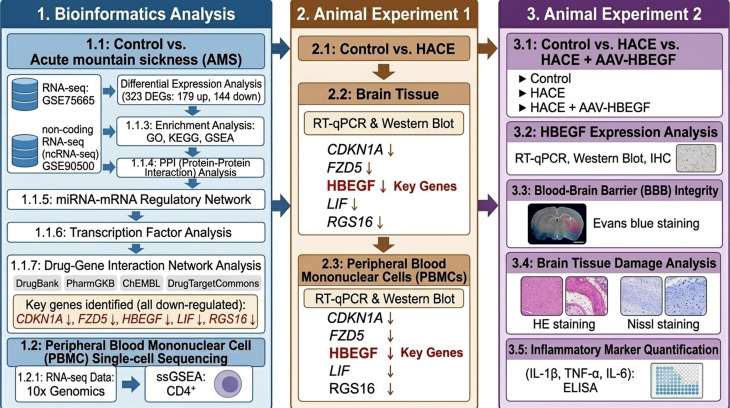
The flow diagram of this study presents a systematic strategy that combines bioinformatics analysis with experimental validation to identify and verify genes associated with brain tissue inflammation in Acute mountain sickness (AMS).

## 1 Introduction

Humans who living, working, or climbing at high altitudes face increased risk of altitude-related illnesses, including acute or chronic mountain sickness (AMS), high-altitude pulmonary edema (HAPE), and high-altitude cerebral edema (HACE) (1), each presenting distinct physiological challenges and health risks (2). The prevalence of AMS among unacclimatized individuals ranges from 40% to 90%, particularly when the daily ascent exceeds 500 meters up to elevations of around 4,500 meters, depending on altitude and individual susceptibility (3). HACE represents a severe and potentially life-threatening progression of AMS, occurring in approximately 0.5%–1% of individuals at elevations between 4,200 and 5,500 meters, with the incidence increasing to 30%–50% at altitudes between 5,500 and 8,000 meters (4, 5). Clinically, HACE manifests with severe headache, ataxia, altered mental status, and, in severe cases, coma or death (6, 7). The pathophysiology of HACE remains incompletely understood, but disruption of the blood–brain barrier, microvascular leakage, and neuroinflammation are thought to play central roles in disease onset and progression (8, 9). AMS and HACE represent a continuum of hypoxia-induced brain injury at high altitude (10). AMS is considered the prodromal stage, whereas HACE is regarded as its severe complication (11). The key difference between the two conditions lies in the degree of cerebral edema and the severity of neurological impairment—AMS is characterized by mild, reversible disturbances of brain function, while HACE involves severe and potentially fatal brain injury (12, 13). In clinical practice, early recognition of AMS and prompt interventions (such as rest, supplemental oxygen, or descent to lower altitude) are critical to preventing progression to HACE (14).

Accumulating evidence suggests that inflammatory processes contribute significantly to the development of HACE (15). Hypobaric hypoxia can induce the release of pro-inflammatory cytokines, activation of endothelial cells, and recruitment of immune cells, ultimately increasing vascular permeability (16). These immune responses may lead to cerebral fluid accumulation and tissue damage. Monocyte subsets dynamically respond to hypobaric hypoxia, with CD16^+^ intermediates expanding during acute exposure and contributing to cytokine storms (17). This may explain the efficacy of immunomodulatory therapies (e.g., dexamethasone) in AMS prevention (18, 19). While previous studies have identified changes in certain inflammatory mediators during acute mountain sickness, the comprehensive molecular regulatory networks underlying inflammation-related gene expression in HACE have not been fully elucidated (20).

Although direct sampling of brain tissue is not feasible in clinical or field settings, peripheral blood transcriptomics has emerged as a valuable and minimally invasive approach to studying systemic immune responses that are intimately linked to neuroinflammatory processes (21). Peripheral blood and the brain are not isolated compartments: under hypoxic and inflammatory conditions, circulating immune cells can be recruited to and infiltrate the central nervous system, where they contribute to blood–brain barrier disruption, neuroinflammation, and cerebral edema formation (22, 23). High-throughput sequencing technologies now allow for systematic characterization of gene expression changes in complex diseases (24). Bulk transcriptome analysis can identify differentially expressed genes (DEGs) between disease and control states, while non-coding RNA profiling can uncover upstream regulatory elements, such as microRNAs (miRNAs), that modulate key gene expression (25). Furthermore, single-cell RNA sequencing (scRNA-seq) provides unprecedented resolution in identifying the specific immune cell populations responsible for pathological processes (26, 27). Integrative analysis of these datasets, combined with protein–protein interaction mapping, transcription factor prediction, and drug–gene interaction exploration, offers an opportunity to reveal novel molecular targets and therapeutic strategies (28, 29).

In this study, we integrated bulk RNA-seq, non-coding RNA-seq, and scRNA-seq datasets from public repositories to investigate inflammation-related molecular mechanisms in HACE. We first identified DEGs between AMS/HACE patients and healthy controls, with a focus on inflammation-related genes. We then explored their functional pathways, regulatory networks involving miRNAs and transcription factors, and potential pharmacological modulators. Finally, using single-cell transcriptomics and deconvolution analysis, we determined the immune cell types most relevant to these inflammation-related genes. Key computationally identified genes were further validated through *in vivo* experiments using a mouse HACE model, including analyses of gene and protein expression, and neuroinflammatory cytokine levels in brain tissue. Our findings provide a comprehensive multi-omics perspective on the inflammatory mechanisms of HACE and identify potential molecular targets for therapeutic intervention.

## 2 Methods

### 2.1 Data sources

All datasets analyzed in this study were obtained from publicly available databases. The RNA-seq dataset GSE75665 was retrieved from the Sequence Read Archive (SRA, https://www.ncbi.nlm.nih.gov/sra) and contains 20 samples, including AMS patients and healthy controls sampled at both low and high altitudes. For the present analysis, only samples collected at high altitude (five AMS and five controls) were included. The peripheral blood non-coding RNA-seq dataset GSE90500, obtained from the Gene Expression Omnibus (GEO, https://www.ncbi.nlm.nih.gov/geo/), comprises 22 samples, with 11 AMS patients and 11 healthy controls, all of which were included in the analysis. Single-cell RNA sequencing data of peripheral blood mononuclear cells (PBMCs) were obtained from 10x Genomics official test datasets pbmc10K and pbmc8K, both derived from healthy individuals. A total of 201 inflammation-related genes were collected from previously published literature (30). It should be noted that altitude exposure may vary among participants in the included datasets, which could influence the degree of hypoxic stress and inflammatory responses. Additionally, potential differences in genetic background among subjects may introduce heterogeneity in susceptibility to altitude-related illnesses and gene expression patterns.

### 2.2 Raw data processing and differential expression analysis

The raw sequencing data of GSE75665 were downloaded from the Sequence Read Archive (SRA) using prefetch (v3.1.1) and converted from SRA to FASTQ format with fastq-dump. The resulting FASTQ files were aligned to the GRCm38 reference genome using STAR (v2.7.11b) to generate gene-level count data for downstream analyses. Differential expression analysis for GSE75665 was performed using the DESeq2 R package (v1.46.0) (31), and genes with p < 0.05 and |log_2_ fold change| > 1 were considered significantly differentially expressed between AMS patients and controls. For the non-coding RNA-seq dataset GSE90500, differential expression analysis was conducted using the limma R package (v3.62.2) (32), with significantly differentially expressed ncRNAs identified using thresholds of p < 0.05 and |log_2_ fold change| > 0.5.

### 2.3 GO, KEGG, and gene set enrichment analysis

Ranked gene lists from differential expression analysis were subjected to GSEA using the clusterProfiler package (v4.12.6) (33). Parameters were set as eps = 0, minGSSize = 10, and maxGSSize = 1000. The top three significantly enriched pathways in each group were visualized using GseaVis (v0.1.0) (34). The intersection between DEGs and inflammation-related genes was subjected to Gene Ontology (GO) and Kyoto Encyclopedia of Genes and Genomes (KEGG) enrichment analysis using clusterProfiler (v4.14.4) (33), with p < 0.05 considered significant.

### 2.4 Protein–protein interaction analysis

The STRING database (https://string-db.org/) serves as a comprehensive platform integrating various types of protein association information. To investigate the relationship between genes with overlapping interactions between differentially expressed genes and inflammation-related genes, we conducted protein interaction analysis using STRING database on these shared genes. The visualization was performed using the ggraph (v2.2.1) (35) R package.

### 2.5 miRNA–mRNA regulatory network

StarBase (ENCORI, https://rnasysu.com/encori/) is a comprehensive RNA interaction analysis platform that integrates data from high-throughput sequencing technologies such as CLIP-seq and degradome-seq, enabling researchers to analyze regulatory networks involving miRNAs, degradome RNAs, and RBP RNAs. This study identified non-coding RNAs (ncRNAs) that can regulate key genes by extracting differentially expressed miRNAs from the GSE90500 dataset. The regulatory network was then visualized using ggraph (v2.2.1) (35).

### 2.6 Transcription factor analysis

To investigate the transcriptional regulatory network of the five key genes, all transcription factors and their regulatory relationships were obtained from the TRRUST database (https://www.grnpedia.org/trrust/). Transcription factors predicted to regulate the five key genes were then selected, and the resulting regulatory network was visualized using the ggraph R package (v2.2.1) (35).

### 2.7 Drug–gene interaction network analysis

The Drug–Gene Interaction database (DGIdb, https://dgidb.org/) integrates drug–gene interaction information from multiple resources, including DrugBank, PharmGKB, ChEMBL, and DrugTargetCommons. These interactions may involve drugs affecting gene expression through genomic interactions or modulating the activity of gene products. To identify potential drugs that could regulate the expression or function of the key genes, all candidate drug–gene interactions were retrieved from DGIdb, and the interaction networks were visualized using network-based graphical methods.

### 2.8 Single-cell data analysis

Single-cell RNA sequencing (scRNA-seq) data of peripheral blood mononuclear cells (PBMCs) from healthy individuals were obtained from the 10x Genomics official datasets. Raw data were imported into the R environment using the DropletUtils R package (v1.24.0) (36). Low-quality cells were filtered based on total counts, the number of detected features, and the proportion of unique molecular identifiers (UMIs) mapped to mitochondrial genes. Cells were filtered based on the following criteria: cells with fewer than 200 detected genes or more than 5,000 genes were excluded, and cells with >10% mitochondrial gene expression were removed to eliminate low-quality or stressed cells. Additionally, doublets were minimized by excluding cells with abnormally high UMI counts. Subsequent analyses included normalization, highly variable gene detection, batch effect correction, dimensionality reduction, clustering, and cell type annotation using established marker genes.

### 2.9 Single-gene enrichment analysis

Single-sample Gene Set Enrichment Analysis (ssGSEA) is an extension of traditional GSEA that evaluates gene set enrichment at the individual sample level rather than between groups, allowing a more detailed assessment of specific gene set expression in each sample. Gene expression values for each sample were rank-normalized, and the ranks were used to compute empirical cumulative distribution functions (ECDFs) for each gene set, thereby reducing the influence of expression level differences and enabling comparability across samples. In this study, ssGSEA was performed using the GSVA R package (v2.0.5) (37) with the key genes defined as the gene set, and the results were visualized using t-distributed stochastic neighbor embedding (t-SNE) plots to identify the cell types predominantly expressing the key genes. The CIBERSORT algorithm (v0.1.0)was used with PBMC single-cell data as a reference to estimate immune cell proportions in GSE75665 samples.

### 2.10 Animal model of hypobaric hypoxia–induced HACE

Male C57BL/6J mice (8 weeks old, 20–22 g; n = 18) were housed under specific pathogen-free conditions with a 12-h light/dark cycle and ad libitum access to food and water. All mice were used in accordance with the approved protocol by the Institutional Animal Care and Use Committee. All animal experimental designs were strictly conducted in accordance with Qinghai Provincial People’s Hospital Province Laboratory Animal Management Measures and were approved by the Ethics Committee of Qinghai Provincial People’s Hospital. To establish a HACE model, mice were exposed to hypobaric hypoxia (HH) environment to induce HACE. Hypobaric hypoxia was achieved using a controlled chamber simulating an altitude of approximately 6,000 m (barometric pressure ~380 mmHg), with a gradual ascent rate over 30 minutes and continuous exposure for 72 hours depending on experimental design. And overexpression of HBEGF in mice was achieved via adeno-associated virus AAV-HBEGF. The AAV vector used in this study was AAV9 carrying the HBEGF coding sequence under the control of the CMV promoter. The viral titer was approximately 1×10^12^ vg/mL, and mice received intravenous administration as described. Mice were randomly assigned to three groups (n = 6 per group): Control group (mice maintained under normoxic conditions); HACE group (mice exposed to hypobaric hypoxia); and HACE + AAV-HBEGF group (mice injected with AAV-HBEGF and subsequently exposed to hypobaric hypoxia).

After the exposure period, mice were euthanized, and blood samples were collected via cardiac puncture. Peripheral blood mononuclear cells (PBMCs) were isolated using density gradient centrifugation with Ficoll-Paque PLUS (GE Healthcare, USA) according to the manufacturer’s instructions. Briefly, blood was diluted 1:1 with PBS, layered over Ficoll, and centrifuged at 400 × g for 30 min at room temperature. The mononuclear cell layer was carefully collected, washed twice with PBS, and resuspended in appropriate medium. Monocytes were further purified from PBMCs using a mouse monocyte isolation kit (negative selection, Miltenyi Biotec, Germany) according to the manufacturer’s instructions, yielding >90% purity as confirmed by flow cytometry (CD11b^+^Ly6C^+^).

### 2.11 Reverse transcription quantitative real-time polymerase chain reaction

Total RNA was extracted from mouse brain tissues and monocyte of the Control and HACE groups using TRIzol™ Reagent (Thermo Fisher Scientific, USA) according to the manufacturer’s protocol. RNA concentration and purity were determined using a NanoDrop spectrophotometer, and samples with an A260/A280 ratio between 1.8 and 2.0 were considered acceptable. Subsequently, 1 μg of total RNA was reverse transcribed into cDNA using the PrimeScript™ RT reagent Kit with gDNA Eraser (Takara Bio, Japan). Quantitative PCR was performed using SYBR™ Green I dye (Thermo Fisher Scientific, USA) on a real-time PCR system (ABI 7500, Thermo Fisher Scientific, USA) with specific primers for HBEGF (forward: 5’-TGGAGATGAAGGTGGTGTTG-3’; reverse: 5’-GGTGATGTTGATGGTGGTGA-3’), CDKN1A (forward: 5’-CCGTGGACAGTGAGCAGTT-3’; reverse: 5’-CCAATCTGCGCTTGGAGTGA-3’), FZD5 (forward: 5’-ATCCTCCGAGAGTTCTGTCCTTGG-3’; reverse: 5’-TCGTCTCCTTCTTCCCTTTGCCT-3’), LIF (forward: 5’-ATTGTGCCCTTACTGCTGCTG-3’; reverse: 5’-GCCAGTTGATTCTTGATCTGGT-3’), and RGS16 (forward: 5’-TGCCACTACCAGTTGCTTCG-3’; reverse: 5’-CTTGAGGAAGCGCGGATAGG-3’).The reaction system was 20 μL in volume and consisted of 40 amplification cycles. Lamin B1 was used as an internal control, and its stability under hypoxic conditions was verified prior to analysis. The relative expression levels were calculated using the 2^−ΔΔCt method. Each sample was assayed in triplicate. No-reverse-transcription (no-RT) controls and no-template controls (NTCs) were included to exclude contamination. Primer specificity was verified by melt curve analysis, and amplification efficiency was evaluated using standard curve analysis, with efficiencies ranging between 90% and 110%.

### 2.12 Western blot

Brain tissues and monocytes were homogenized in RIPA lysis buffer containing protease and phosphatase inhibitors (Thermo Fisher Scientific, USA). Protein concentrations were determined using the BCA Protein Assay Kit (Thermo Fisher Scientific, USA) and normalized for equal loading. Thirty micrograms of protein per sample were denatured with 5×SDS loading buffer, separated by SDS-PAGE (12% resolving gel), and transferred to PVDF membranes (Millipore, USA) using a wet transfer system. Membranes were blocked with 5% non-fat milk at room temperature for 2 h and incubated overnight at 4 °C with primary antibodies against HBEGF (1:500, No.sc-365182, Santa Cruz Biotechnology, USA), CDKN1A(1:1000, ab188224, Abcam, UK), FZD5(1:500, ab75234, Abcam, UK), LIF(1:1000, ab138002, Abcam, UK), and RGS16(1:1000, ab119424, Abcam, UK) and Lamin B1 (0.1 µg/mL, ab16048, Abcam, UK) as the loading control. After washing, membranes were incubated with HRP-conjugated secondary antibodies Goat Anti-Rabbit IgG H&L (HRP)(1:10000, ab6721, Abcam, UK), Goat Anti-Rat IgG H&L (HRP)(1:10000, ab97057, Abcam, UK), and Goat Anti-Mouse IgG H&L (HRP)(1:1000, ab6789, Abcam, UK) for 1 h at room temperature. Protein bands were visualized using enhanced chemiluminescence (ECL) reagents (Thermo Fisher Scientific, USA), and densitometric analysis was performed with ImageJ software.

### 2.13 Immunofluorescence staining

Paraffin-embedded brain sections were deparaffinized and rehydrated. Antigen retrieval was performed using a high-temperature heating method. After three washes with PBS, endogenous peroxidase activity was blocked with 3% H2O2 for 25 minutes at room temperature in the dark. The sections were then blocked with non-immune serum for 20 minutes and incubated overnight at 4 °C with primary antibodies against HBEGF (1:100, sc-365182, Santa Cruz Biotechnology, USA). HBEGF (1:500, No.sc-365182, Santa Cruz Biotechnology, USA) After three washes with PBS, sections were incubated with Alexa Fluor^®^ 488-conjugated Goat Anti-Mouse IgG (H+L) secondary antibody (1:1000, ab150113, Abcam, UK) for 1 hour at room temperature in the dark. After three additional PBS washes, nuclei were counterstained by dropwise addition of DAPI (ZLI-9557, Beijing Zhongshan Golden Bridge Biotechnology Co., Ltd., Beijing, China) for 5 min. Coverslips were mounted with anti-fade mounting medium and sections were stored at 4 °C until imaging. Fluorescence imaging was performed using a fluorescence microscope (Olympus, Tokyo, Japan) at 40x and 200x magnifications. Image analysis and fluorescence intensity quantification were conducted using ImageJ software (National Institutes of Health, Bethesda, MD, USA).

### 2.14 Evans blue assay

Mice were intravenously injected with 2% Evans blue solution (4 mL/kg, Sigma-Aldrich, USA) via the tail vein and maintained for 2 h under normoxic or hypobaric hypoxic conditions. After perfusion with phosphate-buffered saline (PBS) until colorless effluent was observed, brains were harvested, weighed, and homogenized in N,N-dimethylformamide (DMF). Following incubation at 55 °C for 24 h and centrifugation at 12,000 rpm for 30 min, the supernatant was collected and absorbance measured at 620 nm using a microplate reader. Evans blue content was expressed as μg/g brain tissue.

### 2.15 Hematoxylin&Eosin and Nissl staining

To observe histopathological alterations, brains were fixed in 4% paraformaldehyde for 48 h, embedded in paraffin, and cut into 5-μm sections. After deparaffinization and rehydration, sections were stained with hematoxylin for 5 min, differentiated with 1% hydrochloric acid–ethanol for 5 s, and counterstained with eosin for 3 min. Neuronal morphology in the hippocampal CA3 region and cortex was evaluated under a light microscope (Nikon Eclipse 80i, Japan). Neuronal integrity was further examined by Nissl staining. Paraffin sections were deparaffinized, rehydrated, and stained with 0.1% cresyl violet solution at 37 °C for 10 min. After differentiation with 95% ethanol, sections were dehydrated, cleared, and mounted. Neurons in the hippocampal CA3 and cortical regions were assessed, with particular attention to neuronal density, cell body morphology, and Nissl body distribution.

Images were acquired using a fluorescence microscope (e.g., Olympus BX53, Olympus, Japan) equipped with a digital camera (e.g., DP74, Olympus, Japan). Images were captured using a 20× or 40× objective lens under identical exposure settings for all groups to ensure comparability. Exposure time, gain, and illumination intensity were kept constant across all samples within each experiment. For quantitative analysis, images were processed using ImageJ software (National Institutes of Health, USA). Regions of interest (ROIs) were defined consistently across samples based on anatomical landmarks. Background subtraction and threshold settings were applied uniformly across all images. The same analysis pipeline was used for all experimental groups. Quantification was performed by investigators blinded to group allocation to minimize bias.

### 2.16 Measurement of brain water content

Brain water content was determined using the wet-dry weight method. Briefly, after euthanasia, brains were quickly removed and rinsed with cold saline to remove surface blood. The wet weight (WW) of the whole brain or designated brain regions was immediately measured using an analytical balance. The samples were then dried in an oven at 100–105 °C for 24 hours until a constant weight was achieved, and the dry weight (DW) was recorded. Brain water content was calculated using the following formula: (WW-DW)/WW × 100%.

### 2.17 Enzyme-linked immunosorbent assay

To quantify neuroinflammatory cytokines, brain tissues were homogenized in ice-cold PBS containing protease inhibitors and centrifuged at 12,000 rpm for 15 min at 4 °C. Supernatants were collected, and levels of interleukin-1β (IL-1β), tumor necrosis factor-α (TNF-α), and interleukin-6 (IL-6) were measured using commercial ELISA kits according to the manufacturers’ instructions. The following kits were used: Mouse TNF alpha ELISA Kit (ab108910, Abcam, UK), Mouse IL-6 ELISA Kit (ab100713, Abcam, UK) and Mouse IL-1 beta ELISA Kit (ab197742, Abcam, UK). Optical density (OD) was read at 450 nm, and cytokine concentrations were normalized to total protein content determined by the BCA assay.

### 2.18 Statistical analysis

All analyses were performed in R (v4.4.1) and GraphPad Prism 7 (GraphPad Software, La Jolla, CA). Continuous variables were analyzed by t-test or Wilcoxon test as appropriate; categorical variables were analyzed by chi-square test. P < 0.05 was considered statistically significant.

## 3 Results

### 3.1 Differential gene expression analysis between AMS and control groups

**Figure 1:**
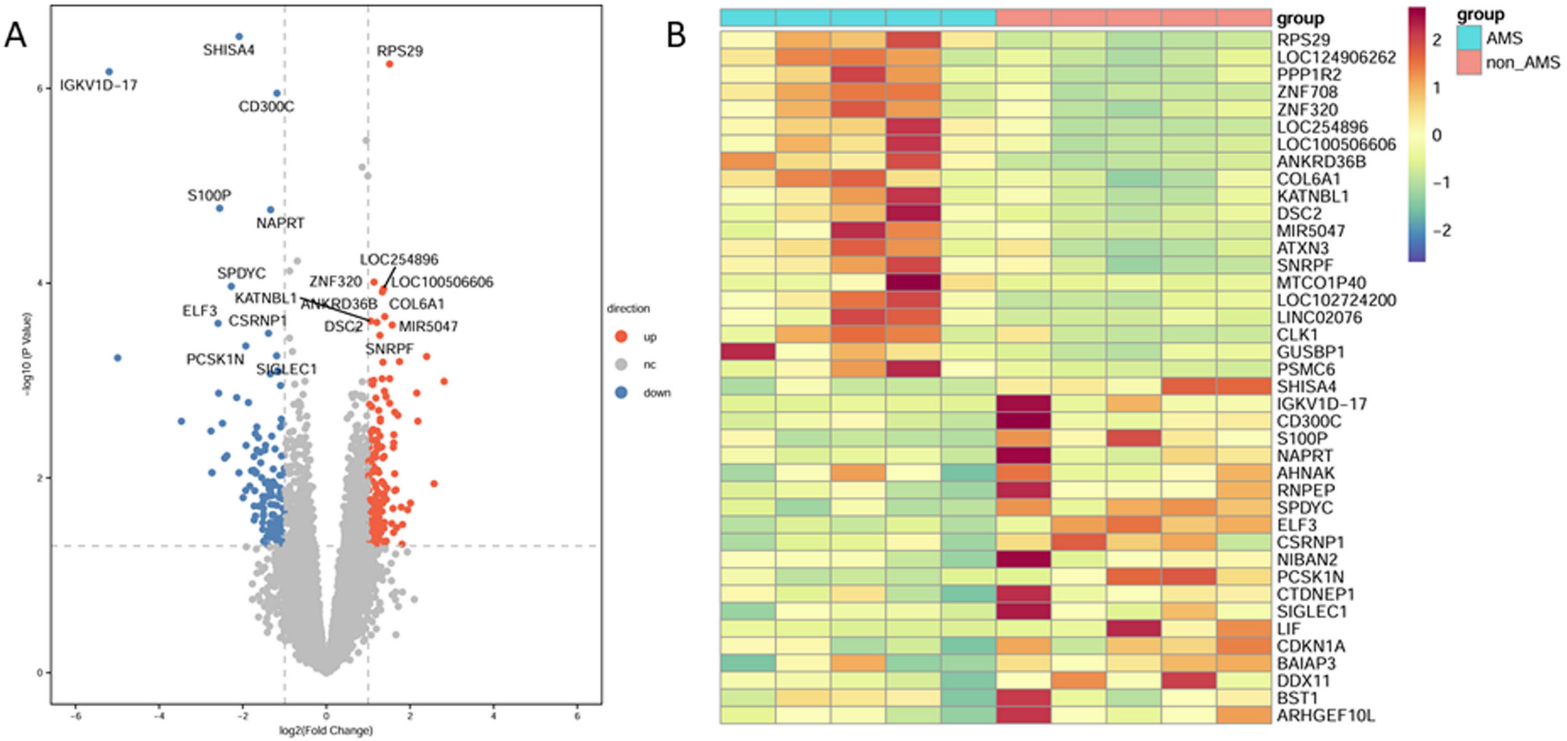
Differential gene expression analysis in acute mountain sickness. **(A)** Volcano plot depicting differential gene expression between AMS patients and controls. **(B)** Heatmap showing the top 20 upregulated and top 20 downregulated genes in AMS. AMS, acute mountain sickness.

Differential expression analysis was performed between AMS patients and healthy controls (**Supplementary Table 1**), and the results were visualized using a volcano plot (**Figure 1A**). Using thresholds of p < 0.05 and |log_2_ fold change| > 1, a total of 323 DEGs were identified, including 179 upregulated and 144 downregulated genes in AMS patients. The top 20 upregulated and top 20 downregulated genes in AMS were subsequently selected to generate a heatmap illustrating their expression levels (**Figure 1B**).

### 3.2 Functional Enrichment Analysis between AMS and control groups

**Figure 2:**
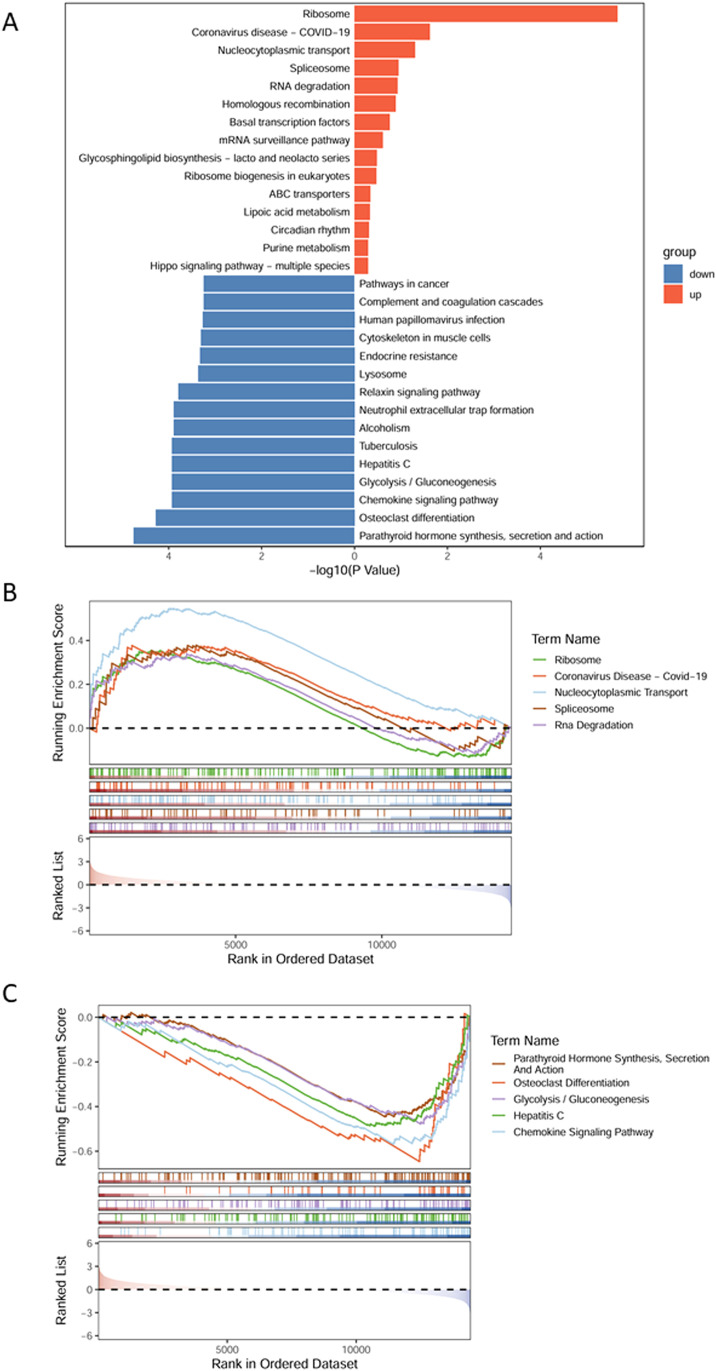
Functional enrichment analysis between AMS and control groups. **(A)** The top 15 enriched pathways in AMS and control groups. **(B)** The top 5 significantly enriched pathways in the AMS group. **(C)** The top 5 significantly enriched pathways in the control group.

To investigate functional changes in blood cells associated with AMS, GSEA was performed using the differential expression results between AMS patients and controls (**Figure 2A**; **Supplementary Table 2**). The analysis revealed that ribosome-related genes and nucleocytoplasmic transport protein-related genes were significantly upregulated in the AMS group compared with controls. Conversely, parathyroid hormone synthesis and secretion, carbohydrate metabolism, and chemokine signaling pathways were significantly downregulated in the AMS group. Additionally, line plots were generated to display the top five significantly enriched pathways in both the AMS (**Figure 2A**) and control groups (**Figure 2C**).

### 3.3 Identification and functional analysis of inflammation-related key genes in AMS

**Figure 3:**
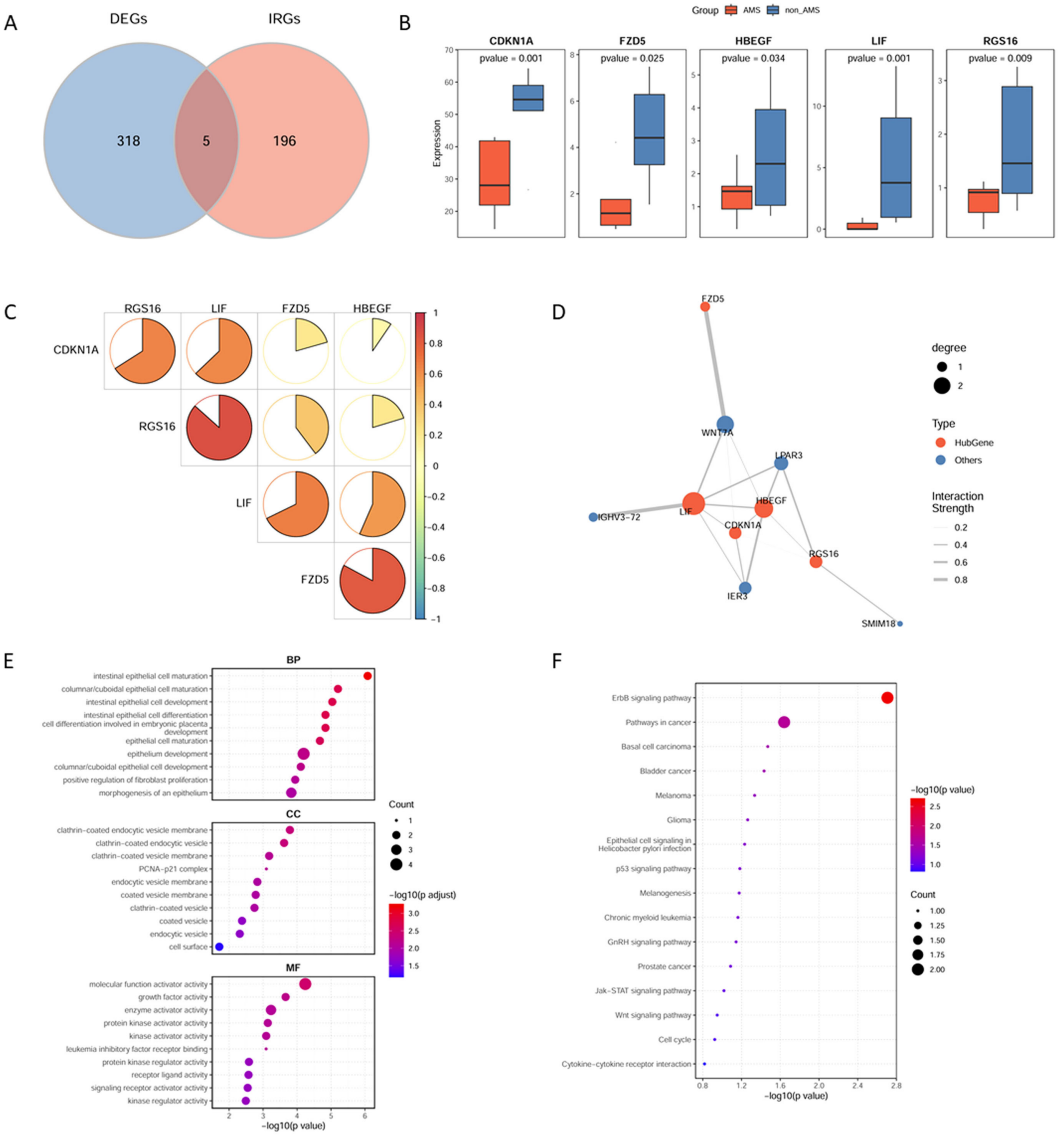
Identification of inflammation-related genes associated with AMS. **(A)** Intersection of differentially expressed genes in AMS and inflammation-related genes. **(B)** Expression levels of key inflammation-related genes between AMS patients and controls. **(C)** Correlation analysis of expression levels among key inflammation-related genes. **(D)** Protein–protein interaction (PPI) network of key inflammation-related genes. **(E)** Gene Ontology (GO) enrichment analysis of key inflammation-related genes. **(F)** Kyoto Encyclopedia of Genes and Genomes (KEGG) pathway enrichment analysis of key inflammation-related genes.

This study focused on identifying inflammation-related factors potentially involved in the pathogenesis of AMS. The intersection of differentially expressed genes in AMS and a curated list of 201 inflammation-related genes yielded 5 key genes (RSG16, FZD5, LIF, CDKN1A, and HBEGF) considered critical inflammation-related candidates (**Figure 3A**). Differential expression analysis showed that the expression levels of these five genes were significantly downregulated in AMS patients compared with controls (**Figure 3B**). PPI analysis of the five key genes revealed strong interactions among LIF, CDKN1A, and HBEGF (**Figures 3C, D**). GO enrichment analysis indicated significant enrichment in gene sets related to epithelial cell growth, differentiation, migration, and endocytosis (**Figure 3E**; **Supplementary Table 3**), while KEGG analysis highlighted significant enrichment of the ErbB signaling pathway (**Figure 3F**; **Supplementary Table 4**).

### 3.4 miRNA regulatory network of inflammation-related key genes

**Figure 4:**
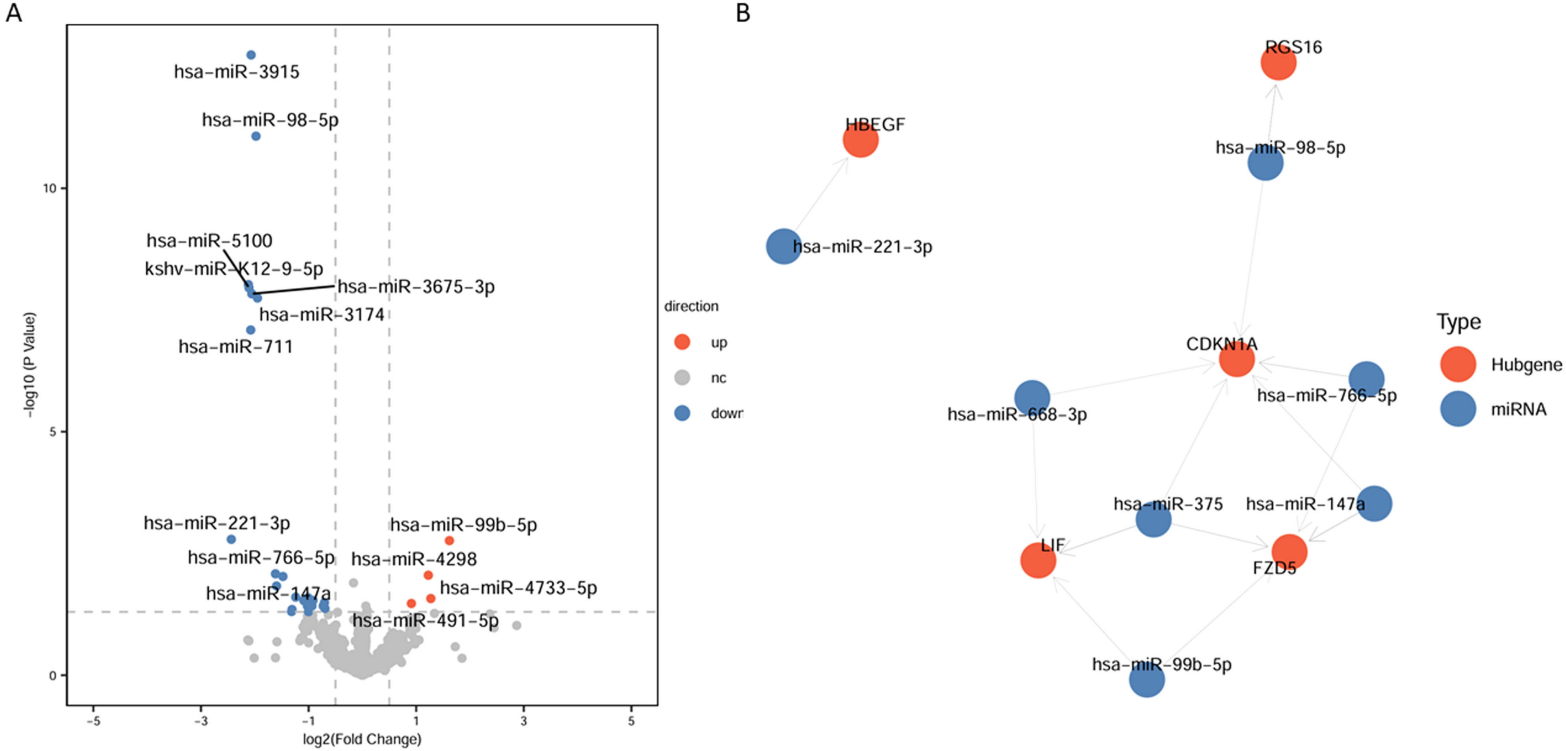
miRNA regulatory network of key genes. **(A)** Volcano plot showing differentially expressed non-coding RNAs between AMS patients and controls. **(B)** Regulatory network of non-coding RNAs and key genes. Red nodes represent key genes, and blue nodes represent non-coding RNAs.

To explore non-coding RNAs potentially regulating the expression of the five key genes, differential expression analysis was performed on the GSE90500 dataset (**Figure 4A**; **Supplementary Table 5**). Using thresholds of p < 0.05 and |log_2_ fold change| > 0.5, a total of 28 differentially expressed non-coding RNAs were identified, including 4 upregulated and 24 downregulated in AMS patients compared with controls. Potential target genes of these 28 non-coding RNAs were retrieved from the starBase database, and those predicted to target the five key genes were selected. The regulatory network was visualized, revealing that hsa-miR-375 can simultaneously regulate LIF, CDKN1A, and FZD5 (**Figure 4B**).

### 3.5 Transcription factor regulatory network and drug–gene interaction analysis of key inflammation-related genes

**Figure 5:**
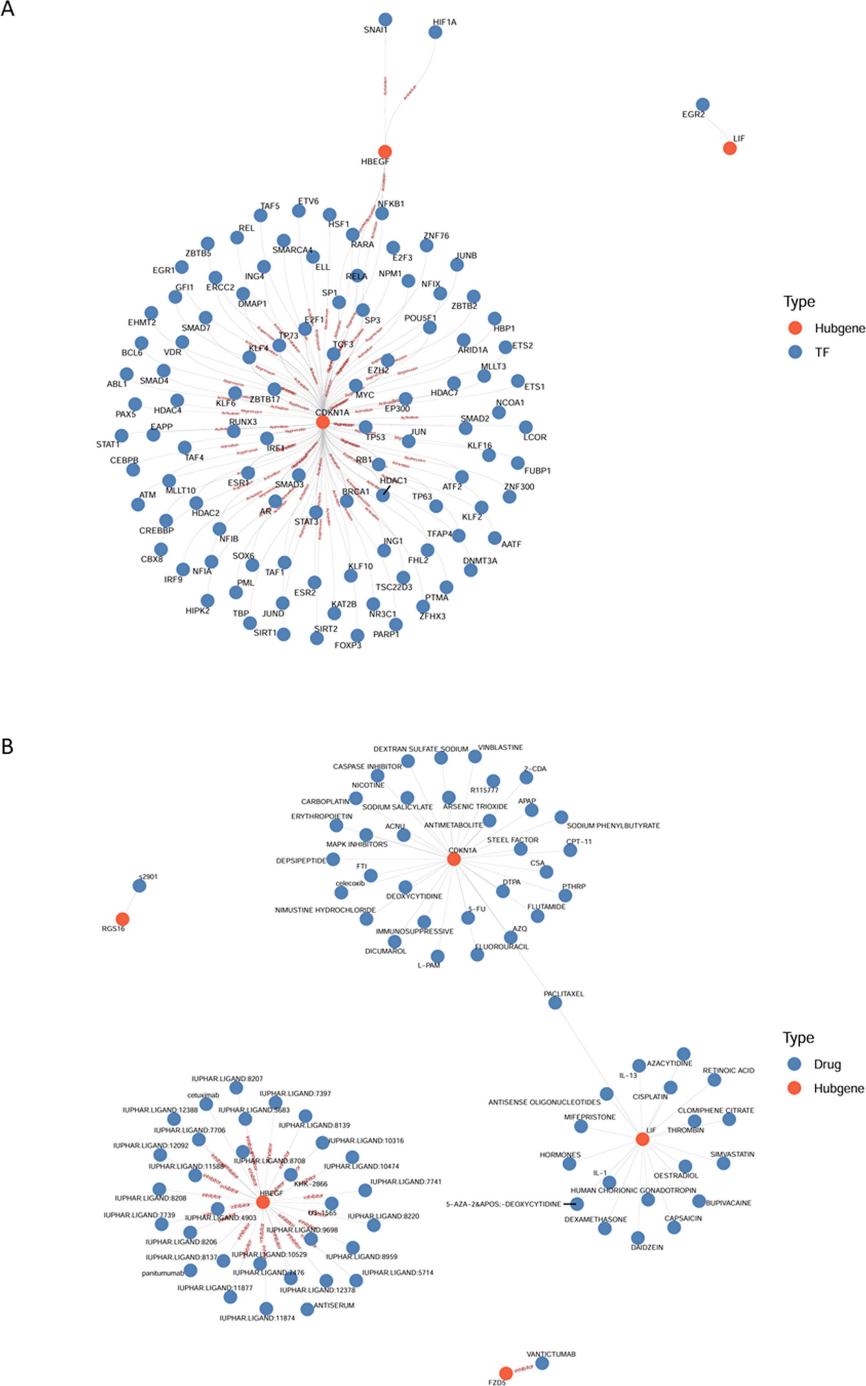
Regulatory network analysis of key genes. **(A)** Transcription factor regulatory network of key genes. Red nodes represent the identified hub genes (CDKN1A, HBEGF, LIF), and blue nodes represent TFs predicted to bind to the promoter regions of these hub genes. Edges indicate potential regulatory interactions between TFs and hub genes. **(B)** Drug-gene interaction network of key genes. TF, transcription factor. Red nodes represent hub genes (RSG16, FZD5, LIF, CDKN1A, and HBEGF), and blue nodes represent candidate drugs or chemical compounds. Edges indicate potential molecular interactions between drugs and their target hub genes.

To investigate the transcriptional regulation of the five key genes, all potential transcription factors were retrieved from the TRRUST database, and those not expressed in the GSE75665 dataset were excluded. The regulatory network was subsequently visualized using network-based graphical methods (**Figure 5A**). Additionally, to explore potential pharmacological regulation, all drugs predicted to interact with the key genes were obtained from the DGIdb database, and the interaction network was visualized (**Figure 5B**). The analysis indicated that HBEGF is negatively regulated by multiple drugs, while other key genes exhibited interactions with drugs, although the precise regulatory effects remain unclear.

### 3.6 Identification of peripheral blood cell types expressing key inflammation-related genes

**Figure 6:**
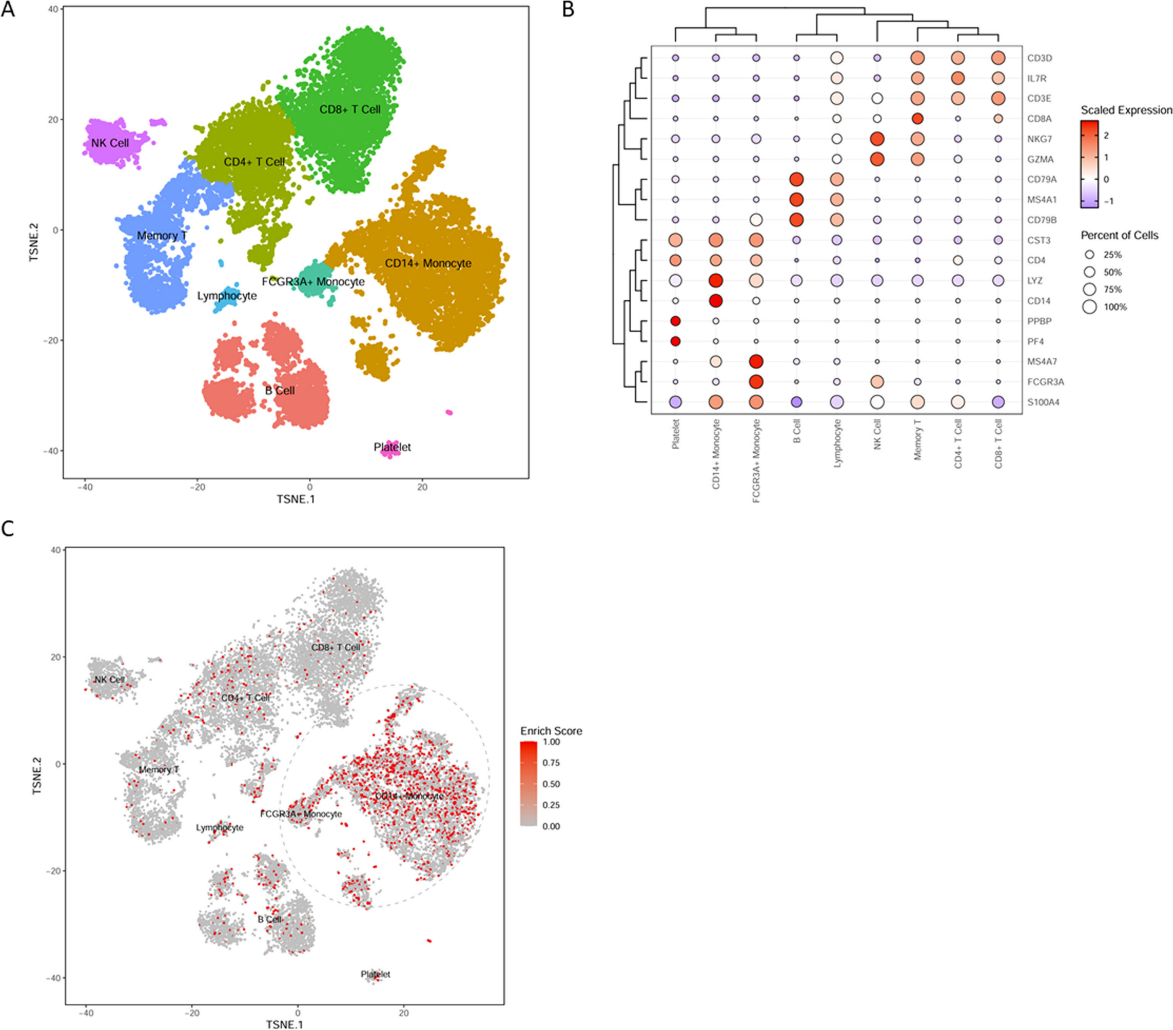
Identification of cells expressing key genes. **(A)** The t-distributed stochastic neighbor embedding (t-SNE) plot visualizing unsupervised clustering of peripheral blood mononuclear cells. **(B)** Bubble plot showing the expression levels of major marker genes used for cell type annotation. The scaled expression (color intensity) and percentage of cells (dot size) expressing canonical marker genes for each identified cell population. **(C)** Distribution of enrichment scores for key gene expression across all cell types. The color gradient (gray to red) indicates the level of hub gene signature enrichment, with red indicating high enrichment.

To determine which peripheral blood cell types predominantly express the five key genes, scRNA-seq data of PBMCs from healthy individuals were downloaded from the 10x Genomics official datasets. The data were subjected to quality control, batch effect correction, dimensionality reduction, clustering, and cell type annotation using established marker genes (**Figures 6A, B**). Single-sample gene set enrichment analysis revealed that the five key genes were primarily expressed in monocytes (**Figure 6C**).

### 3.7 Alterations in peripheral blood cell composition in AMS patients

**Figure 7:**
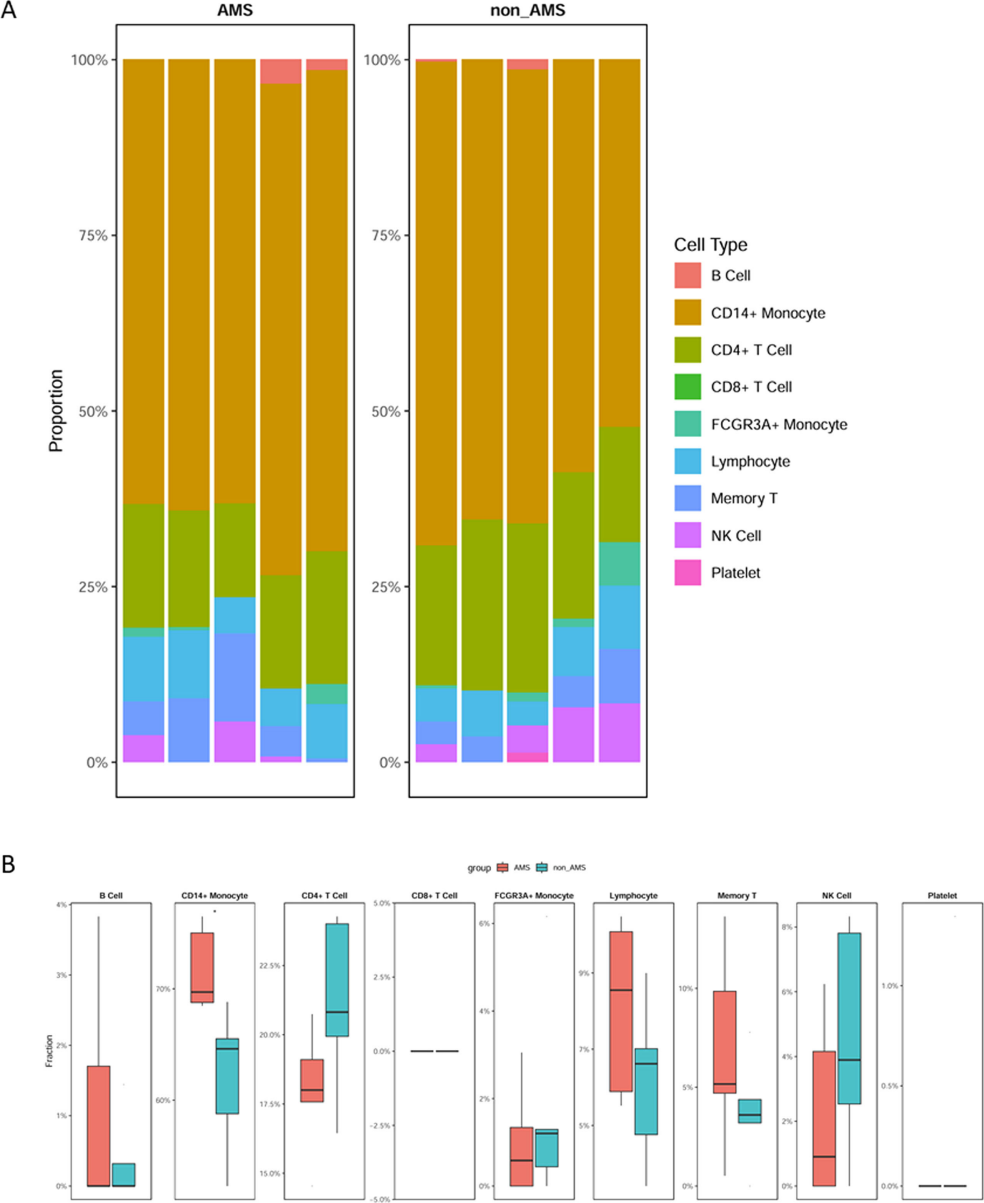
Peripheral blood cell composition analysis in AMS. **(A)** Stacked bar plot showing the relative proportions of major immune cell types in peripheral blood from the AMS (left) and non-AMS (right) groups. **(B)** Boxplots showing the proportion of each cell type between AMS patients and controls.

To investigate potential changes in peripheral blood cell composition in AMS patients, scRNA-seq data of PBMCs from healthy individuals were used as reference, and a deconvolution algorithm was applied to estimate immune cell proportions in the GSE75665 samples (**Figures 7A, B**; **Supplementary Table 6**). The analysis revealed that CD4^+^ monocytes were significantly increased in AMS patients compared with controls.

### 3.8 Downregulation of HBEGF expression in HH-induced HACE mice

**Figure 8:**
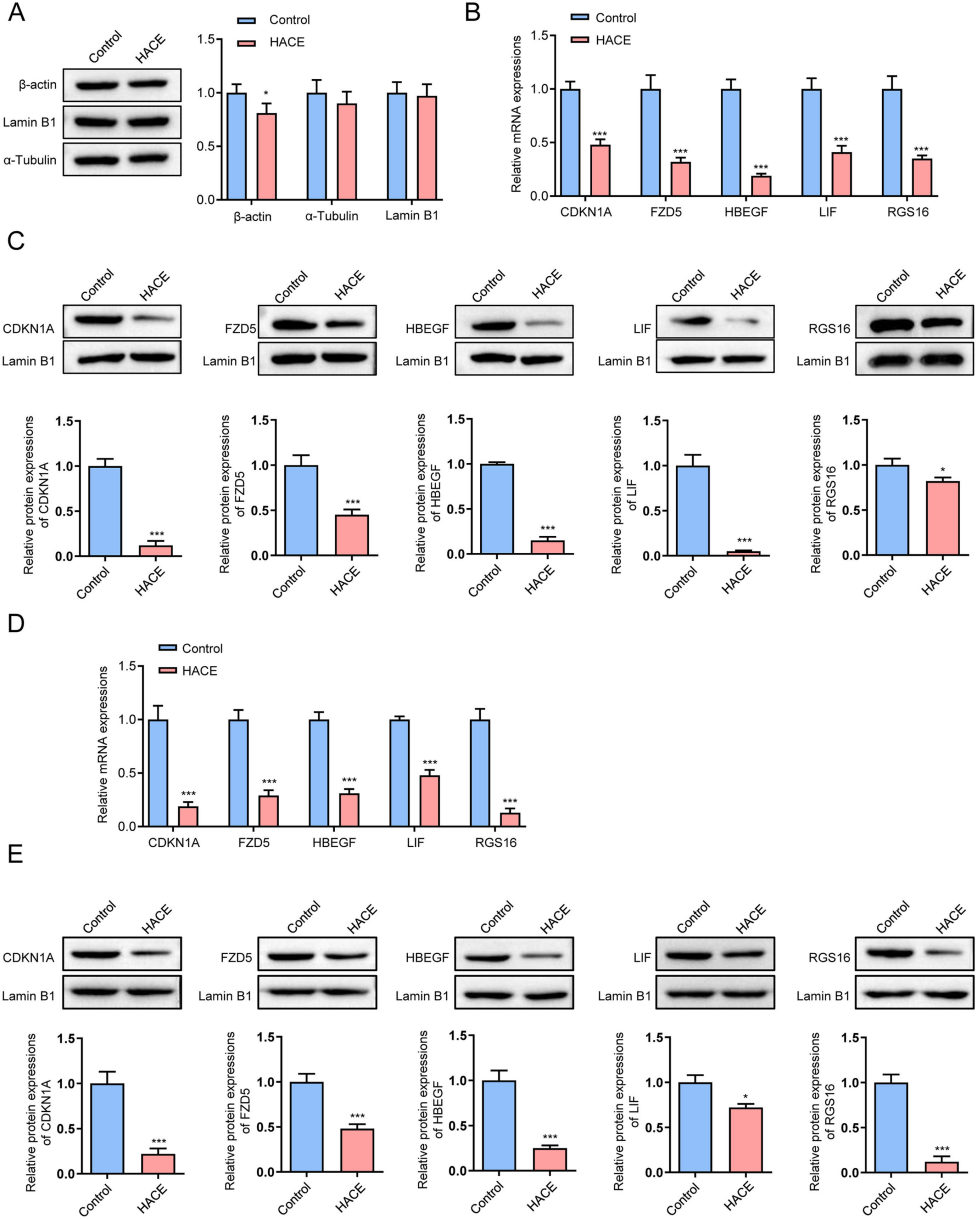
Detection of HBEGF expression. **(A)** Western blot analysis of changes in housekeeping/internal control proteins. **(B)** mRNA expression levels of CDKN1A, FZD5, HBEGF, LIF, and RGS16 in peripheral blood monocytes were determined by RT-qPCR. **(C)** Protein levels of CDKN1A, FZD5, HBEGF, LIF, and RGS16 in peripheral blood monocytes were assessed by Western blot. **(D)** mRNA expression levels of CDKN1A, FZD5, HBEGF, LIF, and RGS16 in brain tissue were determined by RT-qPCR. **(E)** Protein levels of CDKN1A, FZD5, HBEGF, LIF, and RGS16 in brain tissue were assessed by Western blot. All experiments were performed using n = 6 biological replicates per group (Control vs. HACE). Data are presented as mean ± SD. Statistical analysis was performed using Student’s t-test or one-way ANOVA with appropriate *post hoc* tests, as applicable. Significance levels: *P < 0.05, ***P < 0.001 vs. control group.

To investigate the expression profiles of candidate genes in HH-induced HACE, we compared mRNA and protein levels of CDKN1A, FZD5, HBEGF, LIF, and RGS16 between the Control and HACE groups in both peripheral blood monocytes and brain tissue (n = 6 per group). Based on comparative analysis of the stability of the internal control proteins β-actin, α-tubulin, and Lamin B1 in **Figure 8A**, Lamin B1 exhibited the most stable expression. Therefore, Lamin B1 was used as the loading control for all subsequent Western blot experiments. In peripheral blood monocytes, RT-qPCR analysis revealed that the mRNA expression of CDKN1A, FZD5, HBEGF, LIF, and RGS16 was significantly decreased in the HACE group compared with controls (all p < 0.001, **Figure 8B**). Western blot confirmed a marked reduction in the protein levels of CDKN1A, FZD5, HBEGF, and LIF (all p < 0.001), with RGS16 protein also significantly reduced (p < 0.05) in HACE (**Figure 8C**). To further validate whether these changes are consistently observed at the tissue level, we assessed mRNA and protein expression of the five key genes in brain tissue from Control and HACE mice. RT-qPCR demonstrated that CDKN1A, FZD5, HBEGF, LIF, and RGS16 mRNA were all significantly downregulated in the HACE group (all p < 0.001, **Figure 8D**). Western blot similarly confirmed significant reductions in the protein levels of CDKN1A, FZD5, HBEGF, LIF, and RGS16 (p < 0.05, p < 0.001) in HACE brain tissue (**Figure 8E**). Collectively, these findings demonstrate that the five key inflammation-related genes are consistently downregulated in both peripheral blood monocytes and brain tissue in HACE mice, providing cross-compartment validation that supports the biological relevance of the peripheral blood transcriptomic findings identified in human AMS datasets.

### 3.9 HBEGF overexpression protects against HH – induced HACE

**Figure 9:**
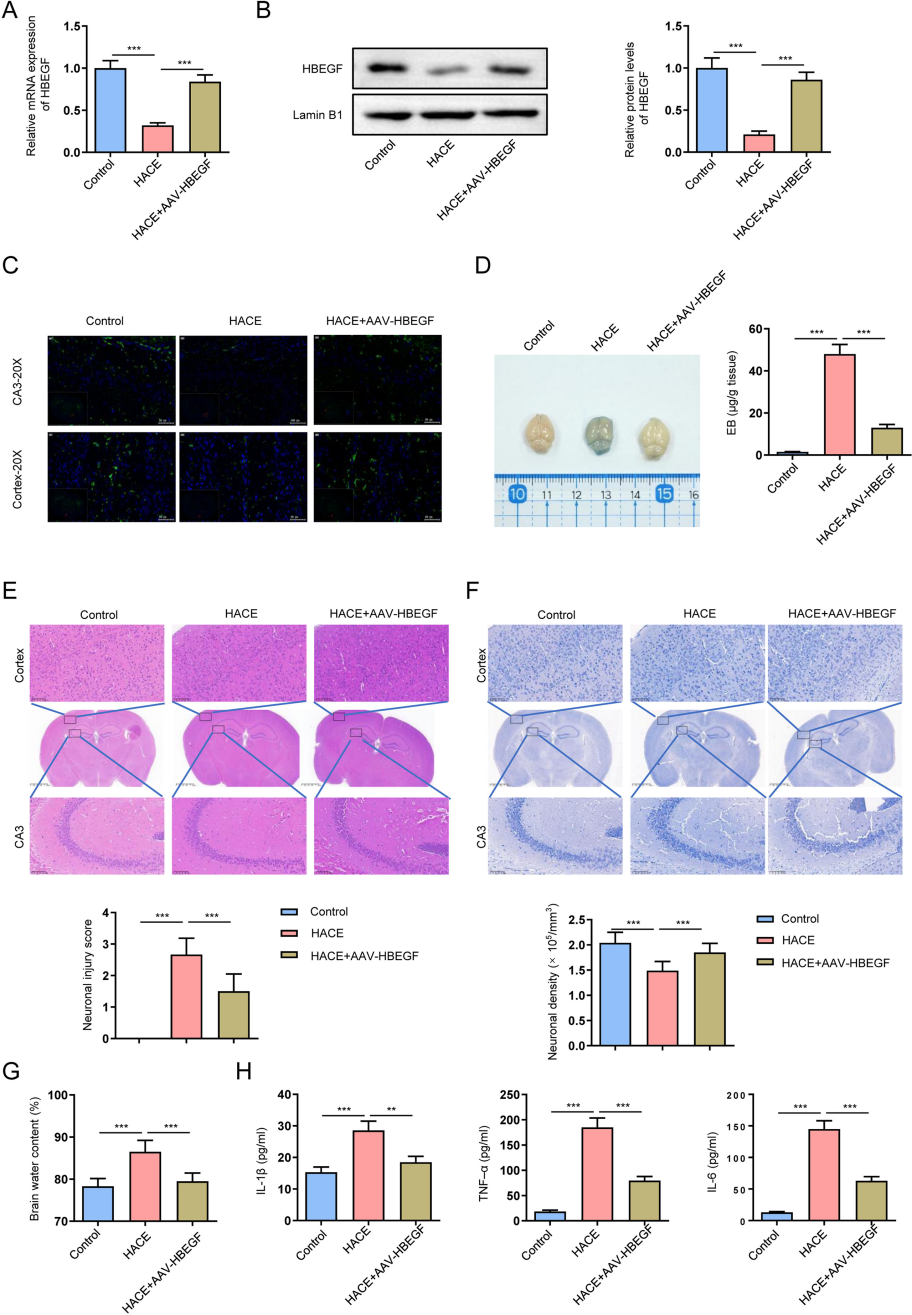
HBEGF overexpression alleviates hypobaric hypoxia–induced HACE in mice. **(A)** HBEGF mRNA expression in Control, HACE, and HACE + AAV-HBEGF groups was measured by RT-qPCR. **(B)** HBEGF protein levels were assessed by Western blot. **(C)** Immunohistochemistry (IHC) analysis of HBEGF expression in the hippocampal CA3 region and cerebral cortex of each group. (scale bars: 50 μm). **(D)** Blood–brain barrier integrity was evaluated by Evans blue extravasation. **(E)** Hematoxylin–eosin (HE) staining showed neuronal morphology in the hippocampal CA3 region and cortex. (scale bars: upper panel 20 μm, middle panel 0.25 mm, lower panel 20 μm). **(F)** Nissl staining assessed neuronal density and structure in the hippocampal CA3 region and cortex. (scale bars: upper panel 20 μm, middle panel 0.25 mm, lower panel 20 μm). **(G)** Brain water content was determined using the wet–dry weight method. **(H)** Levels of proinflammatory cytokines IL-1β, TNF-α, and IL-6 in brain tissues were quantified by ELISA. All experiments were performed using n = 6 biological replicates per group. Data are presented as mean ± SD. Statistical analysis was performed using one-way ANOVA followed by Tukey’s *post hoc* test or Student’s t-test, as appropriate. Significance levels: *P < 0.05, **P < 0.01, ***P < 0.001.

To investigate the role of HBEGF in HH – induced HACE, C57BL/6J mice were assigned to Control, HACE, and HACE + AAV-HBEGF groups. RT-qPCR and Western blot revealed that HBEGF expression in brain tissue was significantly decreased in HACE mice compared with controls, whereas AAV-HBEGF administration effectively restored both mRNA and protein levels (**Figures 9A, B**, p < 0.001). To characterize the spatial distribution of HBEGF in brain tissue, immunohistochemistry (IHC) was performed to assess HBEGF expression in the hippocampal CA3 region and cerebral cortex across all groups. IHC analysis revealed strong HBEGF expression in the CA3 region and cortex of Control mice. HACE exposure markedly reduced HBEGF immunoreactivity in both regions; however, HBEGF fluorescence signals were substantially restored in the HACE + AAV-HBEGF group (**Figure 9C**). Consistently, Evans blue assays demonstrated that HACE induced severe blood–brain barrier (BBB) leakage, while AAV-HBEGF significantly attenuated Evans blue extravasation (**Figure 9D**, p < 0.001). Histological evaluation further confirmed the neuroprotective effects of HBEGF: HE staining (**Figure 9E**) revealed that HACE exposure caused neuronal shrinkage and structural disruption in the hippocampal CA3 region and cortex, whereas these alterations were ameliorated in the HACE + AAV-HBEGF group; similarly, Nissl staining (**Figure 9F**) showed that HACE reduced neuronal density and impaired Nissl body distribution, both of which were preserved by AAV-HBEGF. Functionally, wet–dry weight measurements showed increased brain water content in HACE mice, which was markedly reduced following AAV-HBEGF treatment (**Figure 9G**, p < 0.001). Moreover, ELISA assays demonstrated elevated levels of proinflammatory cytokines (IL-1β, TNF-α, and IL-6) in HACE mice, which were significantly decreased upon AAV-HBEGF (**Figure 9H**, p < 0.01, p < 0.001).

## 4 Discussion

In this study, we performed an integrative multi-omics analysis to elucidate the inflammatory molecular mechanisms underlying HACE. Using bulk RNA-seq, we identified 323 DEGs between AMS patients and healthy controls, of which 5 were inflammation-related genes, LIF, CDKN1A, HBEGF, FZD5, and RGS16. These genes were enriched in biological processes related to epithelial cell growth, differentiation, migration, and endocytosis, with KEGG analysis highlighting the ErbB signaling pathway. Regulatory network analyses revealed hsa-miR-375 as a potential upstream regulator of multiple key genes (CDKN1A, FZD5, and LIF), while transcription factor and drug–gene interaction mapping suggested possible pharmacological targets, particularly for HBEGF. Single-cell transcriptomics and deconvolution analysis further indicated that monocytes are the primary immune cell population expressing these key genes, and that CD4^+^ monocyte levels are significantly elevated in AMS. To enhance biological interpretation and provide cross-species validation, key findings from human datasets were further examined in a hypobaric hypoxia-induced mouse model of HACE, thereby linking peripheral immune alterations to central neuroinflammatory and blood-brain barrier related pathology.

Our results are consistent with prior evidence that neuroinflammation plays a pivotal role in the pathogenesis of AMS. Hypobaric hypoxia has been shown to increase vascular permeability via inflammatory mediator release, ultimately leading to vasogenic cerebral edema (38, 39). The downregulation of LIF and HBEGF observed here aligns with reports that reduced expression of neuroprotective and tissue-repair factors can exacerbate endothelial dysfunction and neuronal injury (40, 41). The enrichment of the ErbB signaling pathway is particularly noteworthy, as this pathway is implicated in regulating inflammatory responses and maintaining epithelial and endothelial integrity (42, 43). Previous experimental studies have demonstrated that inhibition of ErbB signaling can attenuate inflammatory damage, suggesting a potential mechanistic link to AMS pathology.

Importantly, by integrating human peripheral blood transcriptomic data with *in vivo* brain tissue validation, our study provides cross-compartment evidence supporting the systemic-to-central propagation of inflammatory signals in HACE. We demonstrated that all five key genes were consistently downregulated not only in peripheral blood monocytes but also in brain tissue of HACE mice, indicating that peripheral immune alterations are closely mirrored within the central nervous system under hypobaric hypoxia. This finding strengthens the biological relevance of peripheral blood–based transcriptomic analyses and supports their utility as surrogate indicators of neuroinflammatory processes when direct brain sampling is not feasible. *In vivo* experiments provide functional evidence supporting a protective role for HBEGF in HACE. Consistent with our transcriptomic analyses, HBEGF expression was markedly reduced in hypobaric hypoxia–exposed mice, suggesting that its downregulation may contribute to cerebral edema and neuronal injury. Restoration of HBEGF via AAV-mediated overexpression not only attenuated brain water accumulation and preserved blood–brain barrier integrity, but also mitigated neuronal damage in the hippocampal CA3 region and cortex, as demonstrated by HE and Nissl staining. Furthermore, HBEGF overexpression significantly reduced the levels of proinflammatory cytokines IL-1β, TNF-α, and IL-6, indicating that HBEGF can suppress neuroinflammatory responses (44, 45). These findings suggest that HBEGF exerts dual protective functions in HACE, combining anti-inflammatory activity with tissue repair and neuroprotection. Collectively, our data support the notion that HBEGF plays a key mechanistic role in modulating the pathological processes of high-altitude cerebral edema and may represent a promising target for therapeutic intervention.

The identification of hsa-miR-375 as a common regulator of LIF, CDKN1A, and FZD5 adds a novel layer to the understanding of post-transcriptional regulation in AMS. While miR-375 has been implicated in inflammatory and hypoxic responses in other contexts, its role in high-altitude illness has not been reported (46, 47). Similarly, our drug–gene interaction analysis, combined with *in vivo* validation, suggests that pharmacological modulation of HBEGF and other key genes may be a promising therapeutic strategy, warranting further preclinical investigation. Importantly, beyond HBEGF, several additional drug gene interactions identified in DGIdb converge on signaling nodes associated with receptor tyrosine kinase activity and inflammatory modulation, suggesting that multi-target or pathway level intervention strategies may be more effective than single-agent approaches in mitigating hypoxia-induced neurovascular injury. These results collectively highlight ErbB-associated signaling and growth factor mediated pathways as particularly promising candidates for future therapeutic exploration.

By integrating bulk and single-cell transcriptomics, our study provides a more precise cellular context for inflammation-related gene expression in AMS. The finding that monocytes are the predominant source of these key genes, coupled with the increased abundance of CD4^+^ monocytes in AMS patients, suggests that targeted modulation of monocyte-mediated inflammation may help mitigate disease severity (48, 49). The prominence of CD4^+^ monocytes in AMS aligns with their hypoxia-driven pro-inflammatory phenotype (50–52). These cells may initiate neuroinflammation by infiltrating the CNS, as observed in murine HACE models, and by suppressing protective genes like HBEGF. Furthermore, the regulatory networks we identified offer multiple intervention points, including miRNA-based therapeutics, transcription factor modulation, and small-molecule drugs targeting protein products of the key genes.

This study has several limitations. First, because no transcriptomic datasets specific to HACE are currently available in public repositories, we used publicly accessible datasets from AMS, a well-recognized precursor and risk factor for HACE. Although this approach enables the exploration of early molecular changes potentially relevant to HACE pathogenesis, it also introduces inherent differences between AMS and HACE that should be considered when interpreting our findings. In addition, clinical heterogeneity among participants (e.g., ascent rate, altitude exposure, comorbidities) may have influenced gene expression profiles. Second, while our *in vivo* data support a protective role for HBEGF, additional studies are needed to elucidate its precise molecular mechanisms, including downstream signaling events. Third, although our single-cell analysis provides valuable insights into cell-type specificity, it was performed on PBMCs from healthy individuals rather than HACE patients; thus, the immune landscape in disease conditions may differ. Future work should focus on validating the identified molecular targets in patient-derived samples and experimental HACE models, exploring the therapeutic potential of modulating the ErbB signaling pathway, miR-375 activity, and monocyte function, and assessing HBEGF-based interventions in translational studies. Such efforts may contribute to the development of targeted strategies for preventing and treating HACE in high-altitude environments.

In conclusion, our integrative multi-omics analysis reveals a coordinated inflammatory regulatory network underlying high-altitude cerebral edema (HACE), highlighting monocyte-centered immune dysregulation and the ErbB signaling pathway as key mechanistic axes. By integrating bulk and single-cell transcriptomic analyses of human peripheral blood with experimental validation in a hypobaric hypoxia–induced mouse model, we establish a cross-compartment framework linking systemic immune alterations to central nervous system injury under hypoxic stress. Within this network, five key genes (HBEGF, LIF, CDKN1A, FZD5, and RGS16), together with upstream regulation by miR-375, converge on pathways associated with endothelial function, neuroinflammation, and blood–brain barrier integrity, suggesting that HACE arises from multi-layered transcriptional and post-transcriptional immune interactions rather than isolated molecular events. Importantly, HBEGF emerged as a functionally relevant protective factor, as its restoration *in vivo* attenuated blood–brain barrier disruption, reduced neuroinflammation, and alleviated brain edema. These findings not only provide a mechanistically coherent model for HACE pathogenesis but also generate testable hypotheses for future mechanistic studies and suggest that targeting HBEGF-related signaling, miRNA regulation, or the ErbB pathway may represent promising translational strategies for the prevention and treatment of high-altitude cerebral edema.

## Data Availability

The datasets presented in this study can be found in online repositories. The names of the repository/repositories and accession number(s) can be found in the article/**Supplementary Material**.

## Supplementary material

Supplementary Table 1 Differentially expressed genes in AMS.

Supplementary Table 2 Enriched pathways identified by GSEA in AMS.

Supplementary Table 3 Enriched pathways identified by GO in AMS.

Supplementary Table 4 Enriched pathways identified by KEGG in AMS.

Supplementary Table 5 Differential expression of ncRNAs.

Supplementary Table 6 Immune cell composition estimation in AMS.
