# Supplementary figures and images for: Integrative multi-omics analysis reveals inflammation-related molecular networks in acute mountain sickness
Source: Front Immunol. 2026 May 22;17:1745433. doi: 10.3389/fimmu.2026.1745433 (PMC13237639; doi:10.3389/fimmu.2026.1745433)

Figure 8B

CDKN1A


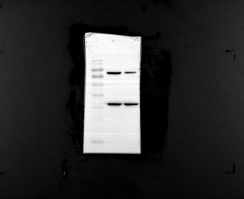


FZD5


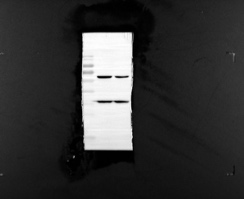


HBEGF


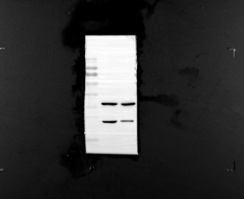


LIF


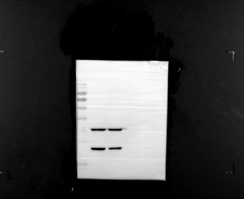


RGS16


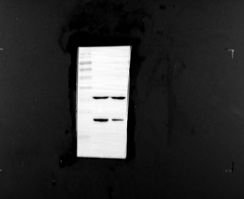


Figure 9B

HBEGF


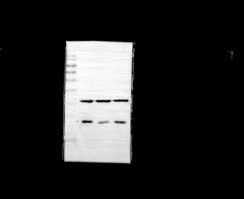

Supplement: Supplementary file 7 [file Table7.docx]
